# Melatonin: A Molecule for Reducing Breast Cancer Risk

**DOI:** 10.3390/molecules23020336

**Published:** 2018-02-06

**Authors:** Alicia González-González, María Dolores Mediavilla, Emilio J. Sánchez-Barceló

**Affiliations:** Department of Physiology and Pharmacology, School of Medicina, University of Cantabria, 39011 Santander, Spain; agonzalez.bq@gmail.com (A.G.-G.), mediavid@unican.es (M.D.M.)

**Keywords:** melatonin, breast cancer risk, antiestrogens, light-at-night, shift work, metalloestrogens, cadmium, obesity

## Abstract

The objective of this article is to review the basis supporting the usefulness of melatonin as an adjuvant therapy for breast cancer (BC) prevention in several groups of individuals at high risk for this disease. Melatonin, as a result of its antiestrogenic and antioxidant properties, as well as its ability to improve the efficacy and reduce the side effects of conventional antiestrogens, could safely be associated with the antiestrogenic drugs presently in use. In individuals at risk of BC due to night shift work, the light-induced inhibition of melatonin secretion, with the consequent loss of its antiestrogenic effects, would be countered by administering this neurohormone. BC risk from exposure to metalloestrogens, such as cadmium, could be treated with melatonin supplements to individuals at risk of BC due to exposure to this xenoestrogen. The BC risk related to obesity may be reduced by melatonin which decrease body fat mass, inhibits the enhanced aromatase expression in obese women, increases adiponectin secretion, counteracts the oncogenic effects of elevated concentrations of leptin; and decreases blood glucose levels and insulin resistance. Despite compelling experimental evidence of melatonin’s oncostatic actions being susceptible to lowering BC risk, there is still a paucity of clinical trials focused on this subject.

## 1. Introduction

### 1.1. Breast Cancer (BC) Risk

Breast cancer is one of the most common neoplasias in women, with about 1.7 million new cases diagnosed worldwide in 2012, representing about 25% of all cancer cases in women, and is the second leading cause of cancer deaths in developed countries [1]. In men, the number of new cases expected to be diagnosed in the USA during 2017 is approximately 2470 [2]. As a result of significant advances in its diagnosis and treatment, the mortality rate for BC is lower than that of its incidence [1]. An increased understanding of the etiology and pathogenesis of BC has allowed the definition of populations who are at high risk for developing this kind of tumor. The National Cancer Institute, in its PDQ Cancer Information Summary published on 28 July 2017, listed the following factors as having “adequate evidence of increased risk of breast cancer”: gender and advanced age, endogenous estrogens, gene mutations (inherited risk), combination hormone therapy (estrogen-progestin), exposure to ionizing radiation, high breast density, obesity and alcohol consumption [3]. As a consequence of the definition of BC risk factors, the search for preventive therapies for this pathology is an expanding field in medical research. Included among the interventions considered to present “adequate evidence of benefit” are chemical treatments (selective estrogen receptor modulators or aromatase inhibitors) and surgical interventions such as prophylactic mastectomy and oophorectomy [3,4]. Obviously, the periodic monitoring of at risk patients is part of the prevention strategies [5].

### 1.2. Melatonin

Melatonin is an indoleamine secreted mainly by the pineal gland with circadian rhythmicity. This molecule exerts regulatory actions on multiple physiological functions. For example, melatonin is involved in the modulation of the neuroendocrine reproductive axis, the metabolism of lipids and carbohydrates, the rhythm of body temperature, the sleep/wake rhythm, the oxidative status, etc. [6,7,8,9]. From numerous experimental studies carried out in rodents it has been demonstrated that melatonin prevents the promotion and growth of both spontaneous and chemically induced mammary tumors [10,11,12,13,14,15]. Furthermore, in vitro, melatonin, at physiological concentrations, inhibits cell proliferation and invasiveness in human breast cancer cells [14,16]. However, despite mounting evidence supporting melatonin actions that relate to BC treatment [17,18], its clinical use in BC therapy, either as the main drug or as an adjuvant therapy in conjunction with other drugs, has only been assayed on a few occasions and these have been mainly related not to the suppression of tumor growth but rather with the relief of symptoms associated with the tumoral process, such as improvement of sleep and life quality [19], treatment of depressive symptoms and anxiety [20], prevention of breast radiation dermatitis [21] or to decrease the toxicity and to increase the efficacy of chemotherapy [22]. It is conceivable that the success of the current hormonal therapies for estrogen receptor positive (ER+) breast cancer, enhanced with the new generations of ER degraders, inhibitors of PI3k, AKT, etc. [23], as well as the effectiveness of new therapeutic strategies for the treatment of triple negative tumors [24], together with the improvement of the classic chemo and radiotherapeutic approaches, are among the reasons that have thus far discouraged the staging of clinical trials to assess the value of melatonin for BC treatment. Another reason might be that what oncologists are actually looking for are drugs which act on molecular targets with high specificity, whereas the antitumoral effects of melatonin are mainly based on its ability to modulate general functions (i.e., circadian rhythmicity, oxidative status, or estrogenic levels) [17,18]. Currently, ten clinical trials examining the therapeutic value of melatonin in BC are listed in the ClinicalTrials.gov. database. Five of them are focused on the relief of symptoms associated with the tumoral process. The remaining five studies examine the therapeutic effects of melatonin either alone (two trials) or as an adjuvant therapy associated to metformin, vitamin D, fluorouracil, doxorubicin or toremifene (three trials) in women already diagnosed with BC, with the results still being analyzed.

### 1.3. Objectives

The aim of this article is to review the evidence that supports the usefulness of melatonin, not for BC treatment, but rather as an adjuvant preventive therapy for BC in several groups of individuals at high risk for this disease. In this review we will attempt to demonstrate that there is ample evidence which suggests that melatonin could be an efficient complement in at least the following situations: (1) where the reduction of BC risk is based on treatment with antiestrogenic drugs; (2) when the risk of BC depends on environmental factors such as chronodisruption due to exposure to light at night (i.e. night shift work) or the exposure to contaminants such as xenoestrogens and, (3) in BC risk cases related to obesity.

## 2. Melatonin as a Possible Adjuvant Therapy When the Reduction of BC Risk is Based on Treatment with Antiestrogenic Drugs

### 2.1. The Role of Estrogens in BC

About 70% of all breast cancers express estrogen-receptors (ER+) and circulating concentrations of estrogens are positively associated with an increased risk of BC in premenopausal women [25]. The particular role of estrogens in the physiopathology of breast cancer explains why chemoprevention using any drug able to antagonize their actions would be taken into consideration [5]. The influence of estrogens on mammary tissue depends on the circulating levels of estradiol and other steroids as well as on their local concentration in mammary tissue.

**Figure 1:**
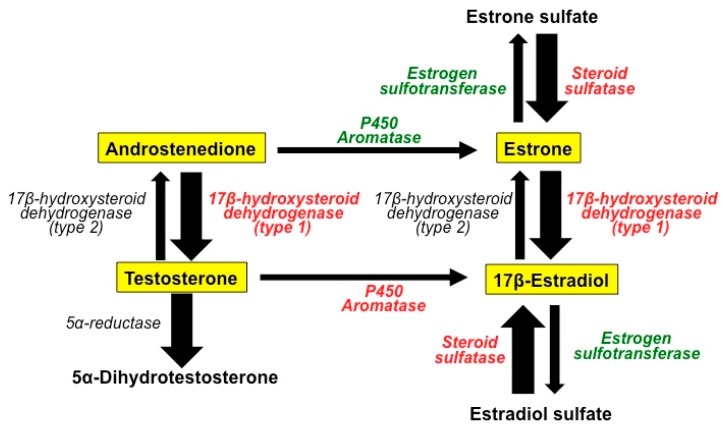
The basic enzymatic mechanisms involved in the transformation of steroids in the normal mammary gland and in breast cancer tissue. Melatonin acts as a selective estrogen enzyme modulators (SEEM) by inhibiting the expression and activity of the enzymes (labeled in red) responsible for the formation of biologically active estrogens from steroids with low biological activity, whereas it increases the expression and activity of the enzymes (labeled in green) involved in the transformation of estrogens into their inactive sulfoconjugates. In breast cancer tissue, the local production of steroids is biased towards production of the more active forms (wide arrows).

While the main source of estrogens in premenopausal women is the ovaries, in postmenopausal women adrenal androgens and sulfated estrogens are the primary circulating steroids, and these steroids are converted into active estrogens by enzymatic processes in the mammary tissue. Figure 1 depicts the basic enzymatic mechanisms involved in the transformation of steroids in the mammary gland [26]. Aromatases are enzymes that transform androgens (testosterone and androstenedione) into estrogens (estrone and 17β-estradiol) [27]. These estrogens, due to the action of estrogen sulfatases and estrogen sulfotransferases may be in two different forms: sulfated or not sulfoconjugated. Estrogen sulfates can serve to form active (non-sulfated) estrogens [28]. Another family of enzymes, the 17β-hydroxysteroid dehydogenases types I and II, catalyzes the conversion of low activity steroids (androstenedione and estrone) into high activity steroids (testosterone and 17β-estradiol respectively) (see Figure 1) [29,30]. Contrary to what happens in normal mammary tissue, in breast cancer, the local production of steroids is biased toward the production of the more active forms (a fact indicated by the thickness of the arrows in Figure 1) [26].

### 2.2. Antiestrogenic Drugs

In the last 20 years, two main families of drugs which are able to interact with the synthesis and actions of the estrogens were developed and are now used for the prevention and treatment of ER+ BC. Selective estrogen receptor modulators (SERMs) are drugs that are able to bind to the ER and act as estrogen antagonists in the uterus and breast. Tamoxifen, a first generation SERM, is probably the most widely used drug in BC prevention. The results of different clinical trials in women classified as having a high risk for BC showed that treatment with tamoxifen (20 mg/day) for 5 years significantly reduced the incidence of ER+ BC, but not of ER− tumors [4,31]. Flushes, as well as increased risk of pulmonary embolism and endometrial cancer are adverse effects described as being associated with treatment with tamoxifen [32]. Raloxifene, a second generation SERM, has also been assayed for BC prevention. A comparative clinical trial between tamoxifen and raloxifene has shown that, although the first appears to be more effective than the second, in terms of reducing BC risk, the undesirable side effects related to tamoxifen, particularly the increased risk of endometrial cancer, are lower in women treated with raloxifene [33].

A second family of antiestrogenic drugs includes the selective estrogen enzyme modulators (SEEMs), that is to say, drugs that do not bind to the ER but instead modulates the activity of the enzymes involved in the conversion of androgens into estrogens, as well as those involved in the interconversion between inactive (conjugated) and active (unconjugated) steroids. Exemestrane and anastrozole are drugs belonging to the SEEM family, and both have antiaromatase properties. Their efficacy in reducing the risk of BC in postmenopausal women has been demonstrated in different clinical trials [5]. Their main negative side effect is a potential increase in osteoporosis as a consequence of estrogen’s depletion [34].

### 2.3. Antiestrogenic Properties of Melatonin

**Figure 2:**
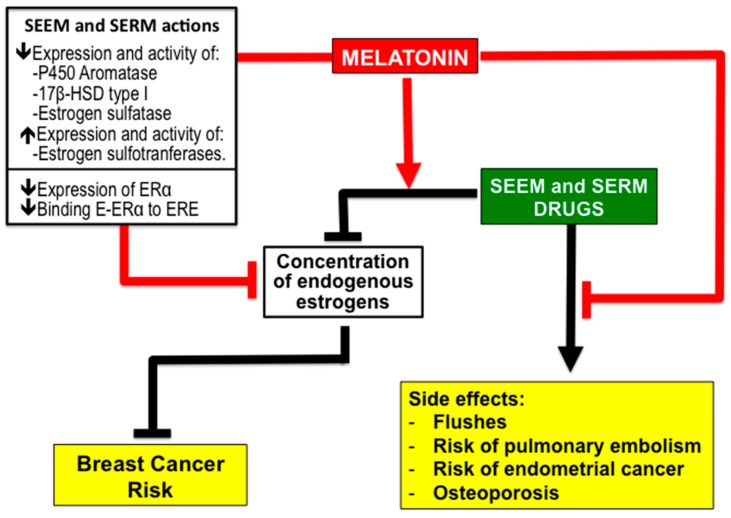
Summary of the evidence supporting the role of melatonin as a possible adjuvant therapy with other selective estrogen enzyme modulators (SEEM) and selective estrogen receptor modulators (SERM) drugs to reduce the risk of breast cancer. Melatonin not only has both SEEM and SERM properties, it also enhances the effectiveness of other antiestrogenic drugs while reducing or preventing their unwanted side effects. Abbreviations: 17β-HSD, 17β-hydroxysteroid dehydrogenase; ERα, estrogen receptor alpha; E, estradiol; ERE, estrogen response element.

Interestingly, melatonin acts as a SEEM and inhibits the expression and activity of P450 aromatase, type I 17β-hydroxysteroid dehydrogenase and estrogen sulfatase, that is to say, the expression of the enzymes responsible for the formation of biologically active estrogens from steroids (androgens and estrogens) with low biological activity (Figure 2). Furthermore, melatonin increases the expression and activity of estrogen sulfotransferases, thus favoring the transformation of estrogens into their inactive sulfoconjugates (Figure 2) [35,36,37,38,39,40,41,42,43]. Melatonin has also been shown not only to possess SEEM properties but also to act as an SERM [35,36,37,38,39,40,41,42,43]. In MCF-7 human breast cancer cells, the melatonin binding to the MT1 and MT2 receptors present in the membranes of these cells [44,45,46] down-regulates the expression of ER-α and inhibits the binding of the estradiol-ER complex to the estrogen response element in DNA (Figure 2) [47,48].

### 2.4. Melatonin Potentiates the Efficiency of SEEM and SERM Drugs and Reduce Their Side Effects

In addition to its simultaneous properties functioning as both a SEEM and a SERM, experimental data has also revealed that melatonin increases the sensitivity of MCF-7 cells to the effects of antiestrogens such as tamoxifen [49], and that pretreatment with melatonin increases the reduction of aromatase expression and the activity of cells exposed to aminoglutethimide, an anti-steroid drug [50]. This evidence supports the idea of using melatonin to enhance the effects of conventional SEEM and SERM drugs (Figure 2).

Furthermore, melatonin could be also useful for reducing or suppressing some of the side effects of conventional antiestrogens (Figure 2). In this regard, it has been demonstrated, in animal models, that melatonin reduces the hepatotoxicity of the aromatase inhibitor letrozole [51]. A recent patent (US8785501; 2014, from Witt-Enderby et al., Duquesne University of the Holy Spirit, USA) reports a tamoxifen-melatonin hybrid ligand for BC treatment. In mice, the administration of the hybrid compound *N*-desmethyl-4-hydroxytamoxifen-melatonin showed anticancer effects without the uterine hyperproliferation observed after treatment with tamoxifen alone. These results have not yet been published in ordinary scientific journals.

Interestingly, the possible association of melatonin with antiaromatases could reduce the risk of osteoporosis described with treatment with this family of drugs. Melatonin promotes osteoblast proliferation and the synthesis of osteoprotegerin, a member of the tumor necrosis factor receptor (TNFR) superfamily. This protein inhibits the differentiation of osteoclasts by preventing the binding of osteoclast differentiation factor (ODF) to receptor activator of NK-κB ligand (RANK) on the differentiating osteoclasts. In this way, melatonin could cause an inhibition of bone resorption and an increase in bone mass [52,53,54,55]. In addition, the osteoclastic activity generates free radicals that induce bone degradation and resorption [56]; melatonin may reduce the osteoclastic activity by neutralizing free radicals and stimulating the activity of antioxidant enzymes [56,57]. The usefulness of melatonin in the treatment of osteoporosis has been analyzed in several clinical trials carried out in postmenopausal women with osteopenia. Administering melatonin to these women (1–3 mg/day for 6–12 months) improved bone mineral density and decreased the risk of fractures [58,59]. The role of melatonin in bone metabolism is also supported by indirect data such as the association between the incidence of hip and wrist fractures and nightshift work (which suppresses melatonin production) in postmenopausal nurses [60], and between the increased incidence of osteoporosis and the decrease of melatonin production with age [61].

A meta-analysis of 51 epidemiological studies comprising more than 150.000 women, carried out by the Collaborative Group on Hormonal Factors in Breast Cancer [62] concluded that hormone replacement therapy (HRT) after menopause was associated with an increased risk of BC. The association of melatonin with conventional HRT (estrogens + progesterone) to prevent the risk of HRT-related BC was patented a few years ago (US8618083; 2013, from Witt-Enderby et al., Duquesne University of the Holy Spirit, USA). The patent documents described experiments carried out on mice, concluding that associating melatonin with HRT significantly increases the latency and decreases the incidence of mammary cancer.

In conclusion, the above-mentioned data strongly supports the possible benefits of the association of melatonin with conventional antiestrogens as well as HRT to reduce the risk of BC, and clinical trials to analyze the efficiency of these cotreatments are encouraged.

## 3. Melatonin for Reduction of BC Risk due to Environmental Factors

The “classical” risk factors for breast cancer, that is to say, genetic mutations, reproductive history, obesity, alcohol consumption, radiation and sedentary lifestyle do not explain all the potential situations of increased risk of mammary carcinogenesis. The link between BC risk and environmental factors is a subject of growing relevance [63]. There is one environmental factor that, in our opinion deserves special consideration: the chronodisruption induced by the exposure to light-at-night (LAN), particularly short wavelength light (i.e., blue-light). The reason is the growing number of individuals involved in nocturnal shift work. A second group of environmental factors related to BC risk includes chemical contaminants present in food, pharmaceutical agents and cosmetics, or due to occupational exposures, etc. [63]; especially interesting are those environmental contaminants with estrogenic properties, known as xenoestrogens.

### 3.1. Melatonin Reduces BC Risk from Exposure to Light-at-Night (LAN) Producing Chronodisruption. The Shift Work

**Figure 3:**
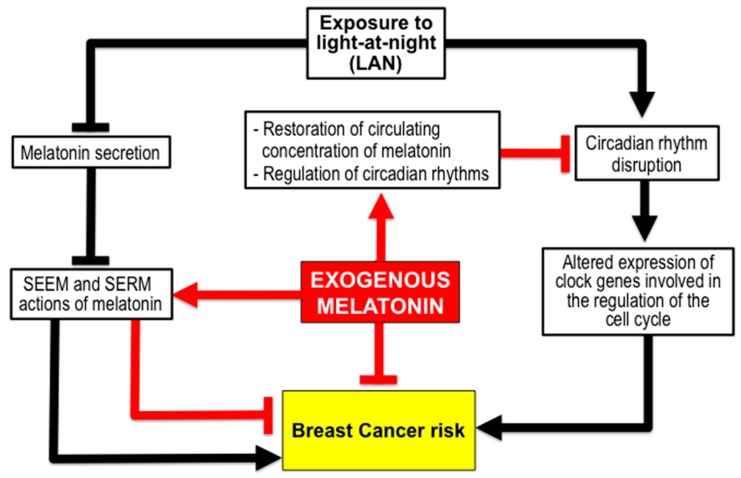
The administration of melatonin could reduce the breast cancer risk associated with chronodisruption caused by exposure to light at night, such as occurs in nocturnal shift workers. The exogenous melatonin restores the decreased circulating levels of the endogenous hormone, caused by nocturnal light, and regulates the function of the circadian system.

Circadian rhythm disruption induces alterations in one hallmark of cancer, cell division, thus contributing to cancer progression, and the pharmacological modulation of proteins related to clock genes is being considered as a possible strategy for cancer treatment [64,65]. Chronodisruption from LAN represents a factor for BC risk that could be reduced by melatonin (Figure 3).

That LAN increases the rate of growth of mammary tumors has been solidly established in experiments carried out in rodents with chemically induced tumors or xenografts of cancer cells [66,67]. Furthermore, mutant mice prone to developing mammary tumors, increased their risk of developing such tumors when exposed to chronically alternating light cycles simulating a shift work [68].

The association between shift work including night work and the increased risk of different kinds of cancer is supported by numerous studies [64,69,70,71], although the mechanisms underlying this relationship are still under study. The risk of BC increases with the number of years devoted to shift work, with an estimated 16% risk increase for every 10 years [71]. The potential impact of shift work in breast cancer has been estimated at a population attributable fraction of 5.7% [72]. Epidemiological studies, despite some controversies, support the relationship between long-time night shift work and an increased risk of breast cancer [63]. The International Agency for Research on Cancer (IARC) classified night shift work that involves circadian disruption as a probable human carcinogen (group 2A) [73]. Based on this classification, the National Board of Industrial Injuries in Denmark has recognized BC as an occupational disease, in women that had been doing night shift work for at least one day per week over 20 or more years. Consequently, these women, mainly nurses and flight attendants, received an economical compensation from the Danish government [74]. There are, however, discrepancies about the carcinogenic effects of nocturnal work, and several epidemiological studies conclude that night shift work has little or no effect on breast cancer incidence [75]. These discrepancies may be related to methodological differences between the studies, due to the complexity of this kind of analysis [63]. As an example of this complexity, a recent publication about a survey carried out in the Canadian province of British Columbia, comprising 30,700 shift workers at 88 companies described ¡more than 400 different shift work systems¡ [76]. To these variables, others which are dependent on the workers’ particular circumstances introduce even more complexity: the age of the women, their reproductive status, the duration of exposure to nocturnal light, the subtypes of breast cancer, etc. The most recent published review on this subject concluded that there is a tendency toward an increased risk of BC among women after 20 or more years of shift work or even after shorter periods involving many consecutive shifts [77]. Interestingly, the authors of this review conclude that: “evidence-based preventive interventions are needed” [77].

The two most widely accepted explanations for the biological effects of LAN in elevated BC risk are: the light-induced inhibition of melatonin secretion, with the subsequent loss of the SEEM and SERM effects of this neurohormone; and alterations to the circadian system (chronodisruption) induced by exposure to nocturnal light [15,39,40,41,42,78,79,80,81,82,83,84].

Regarding the influence of decreased melatonin secretion, a relationship between low plasma melatonin concentration and breast cancer was described as early as 1978 by Cohen et al. [85]; women with ER+ breast adenocarcinomas had nocturnal plasmatic concentrations of melatonin significantly lower than both healthy women and women suffering ER-breast cancer. These authors coined the expression "relative hyperestrogenism" to define the hormonal situation of women with low melatonin secretion, whatever its cause. Postmenopausal women working at night have elevated serum estradiol levels and a significant decrease in urinary excretion of 6-sulfatoxymelatonin (aMT6s), a metabolite of melatonin that serves as an indicator of melatonin secretion [86,87,88].

The cell cycle is controlled by the circadian system, and the progression through its different stages occurs at specific times of the night/day cycle. Although the body’s central clock, situated in the suprachiasmatic nucleus of the hypothalamus is responsible for the synchronization of the entire circadian system, peripheral clocks located in almost all cells assume important regulatory functions such as control of the cell cycle. The coupling between the cellular clocks and the cell cycle is the key to explaining the relationship between chronodisruption and cancer [89,90]; the disruption of the circadian rhythms induced by LAN can cause alterations in the cell cycle and lead to the development of tumors [91,92]. In a recent study [93], carried out with a rat model with ER+ MCF-7 tumor xenografts, LAN not only increased the rate of tumor development but also conferred resistance to tamoxifen therapy as well as to chemotherapeutic treatment with doxorubicin [94]. A putative role for the *CLOCK* gene in the development of breast cancer in shift workers has been proposed [95], and epidemiological studies carried out involving shift working women have shown a relationship between breast cancer susceptibility and polymorphisms in *CLOCK*, *BMAL1*, *BMAL2* and *NPAS2* clock genes [96,97].

The administration of melatonin (1–10 mg/day) to night shift workers has been widely assayed as a way to ameliorate the body’s adaptation to nocturnal work or to lessen insomnia during the day [98,99,100]. However, despite the experimental and epidemiological evidence regarding the role of LAN in mammary carcinogenesis, the benefit of melatonin supplementation in women working at night is still yet to be assessed.

### 3.2. Melatonin and BC Risk due to Exposure to Xenoestrogens like Cadmium (Cd).

**Figure 4:**
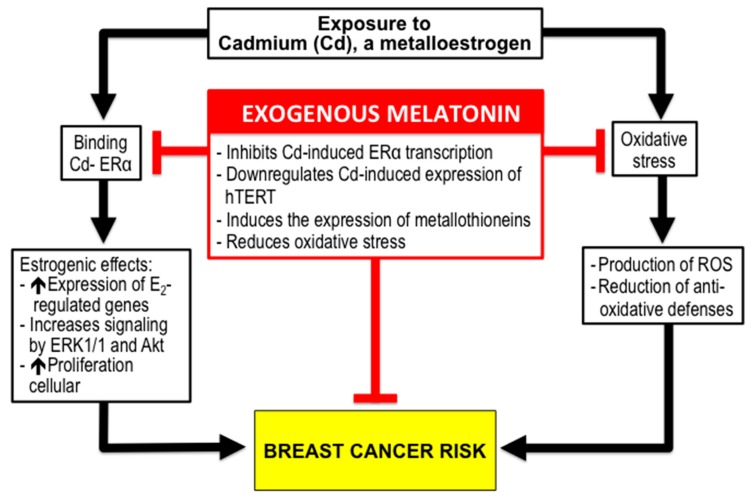
Cadmium, a metalloestrogen, increases the risk of breast cancer by stimulating cell proliferation due to its estrogenic properties, as well as by producing oxidative stress through mitochondrial damage. Melatonin could reduce the risk of breast cancer in individuals exposed to Cd from contaminated food, smoke, or occupational hazards. Melatonin, acts as a specific inhibitor of Cd-induced ERα-mediated transcription in estrogen response elements. Furthermore, melatonin downregulates the hTERT expression induced by Cd and increases the synthesis of metallothioneins, which are proteins involved in Cd detoxification. Moreover, melatonin prevents against Cd-induced oxidative stress.

Among the environmental factors with possible incidence in BC risk, the role of chemical contaminants is a matter of discussion, although there is general agreement that environmental estrogen-like compounds (xenoestrogens) could be related to the onset and development of BC [101]. Currently, more than 160 xenoestrogens potentially involved in BC development have been identified [102]. The term metalloestrogens refers to several metals which are able to bind and activate ERα thus mimicking the actions of endogenous estrogens [103]. In this review, we have focused our interest on the metalloestrogen, cadmium (Cd), for three reasons: it was designated as a Group 1 human carcinogen in 1993 by the IARC [104]; its implication in health outcomes like BC has been recently confirmed by different studies [105,106]; there is consistent data concerning melatonin’s role against Cd’s effects [107] (Figure 4).

Cd is naturally present in soils, sediments, seawater, plants, and animals and is a food-chain contaminant. An individual’s environmental exposure to Cd depends on dietary sources but also from tobacco smoke (a pack of cigarettes contains about 2–4 μg of Cd) [106]. Occupational exposure to Cd is significant in industrial processes such as: mining and processing of zinc, copper and lead ores, smelting, galvanizing, electroplating, the manufacture of pigments/paints/sealants and plastic stabilizers, and manufacturing photovoltaic devices and nickel/cadmium batteries. Due to its long biological half-life Cd accumulates in the body [108]. A survey carried out in the USA comprising the period 1999–2008 revealed a Cd exposure prevalence of 94–98% in the non-smoker population (age range 20–85 years) and 96–99% in smokers [109]. Because of this high exposure prevalence, any increase in risk of disease from Cd would result in a large number of affected individuals [106].

Although the kidneys are the organs most affected by Cd exposure, epidemiological studies have demonstrated that this metal can increase the risk of BC [105,110,111,112,113]. The concentration of Cd in the blood [114] as well as in the urine (a measure of cumulative lifetime Cd) of BC patients is significantly higher than in healthy controls [115,116,117,118], and a positive correlation between Cd content in breast tumor tissue and the histological type of tumor, its size, grading and progesterone receptor status, has been described [115,119]. In Japanese women, elevated dietary intake of Cd has been associated with ER+ breast cancer [120]. Furthermore, it has recently been described that in utero exposure to Cd increases stem/progenitor cells, cell density, and expression of ERα, thereby constituting a risk factor for BC development [121]. There are, however, other studies that showed no association between urinary concentration of Cd and risk for development of BC [122,123].

There are two basic explanations for the effects of Cd on the mammary gland. One, is related to the estrogenic effects of this metal. Cd increases the in vitro proliferation of ER+ breast cancer cells as well as the expression of estrogen-regulated genes, and increases signaling by ERK1/2 and Akt pathways [124,125]. In vivo, Cd activates the genomic and non-genomic ER pathways in mammary glands [126]. The second reason for the damaging effects of Cd is related to its ability to induce oxidative stress. Although Cd does not produce radicals in Fenton type reactions, it induces oxidative stress through the reduction of antioxidative defenses as well as by the production of reactive oxygen species through mitochondrial damage [127].

Melatonin exerts remarkable actions against the estrogenic effect of Cd. In MCF-7 human BC cells, melatonin prevents cell proliferation by acting as a specific inhibitor of Cd-induced ERα-mediated transcription in both, estrogen response elements and AP1-containing promoters [128]. Furthermore, melatonin down-regulates the Cd-induced expression of hTERT, the telomerase subunit main determinant of its enzyme activity [129]. In vivo, melatonin prevents the estrogenic effects of Cd on mice mammary glands and uterus [130]. Melatonin has also been shown to induce the expression of metallothioneins [131]. These are intracellular low molecular weight (6–7 kDa) cysteine-rich proteins ubiquitous in eukaryotes, and are involved in detoxification mechanisms against Cd [127], and have been linked to the prognosis of BC [132]. Moreover, numerous studies have already evidenced melatonin’s role in protecting against Cd-induced oxidative stress [133,134]. This data should serve to recommend the use of melatonin for individuals who frequently encounter Cd, due to occupational hazards, or for those residing in geographic areas with high levels of contamination, as a prophylaxis against the increased risk of BC from exposure to this metalloestrogen.

## 4. Melatonin, Obesity and BC Risk

**Figure 5:**
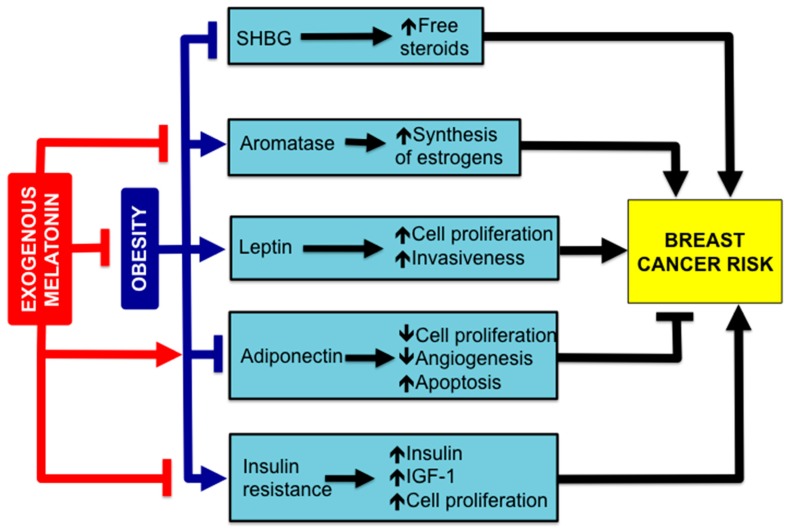
The increased breast cancer risk associated with obesity is related to factors such as: a low concentration of sex hormone-binding globulin (SHBG), which increases the bioavailability of sex steroids; an enhanced expression of aromatase, and the subsequent increase of local synthesis of estrogens; an elevated secretion of leptin by white adipose tissue (WAT) that stimulates cell proliferation; a low concentration of adiponectin, an adipokine that inhibits cell proliferation and angiogenesis and induces apoptosis; the development of insulin resistance, with an elevated insulin blood concentration which induces the release of growth factors (IGF-1), stimulating cell proliferation. Melatonin, stimulates adiponectin secretion; inhibits aromatase expression and prevents insulin resistance. Furthermore, melatonin reduces body weight gain, diminishing obesity.

Epidemiologic studies included in the Women’s Health Initiative Observational Study have concluded that generalized obesity represents an important risk factor for breast cancer among postmenopausal women who have never received HRT, in comparison with slimmer women [135,136,137]. Body weight (BW) gain, as well as a longer period of time spent being obese or overweight are also BC risk factors in these women [138,139]. The association between BW gain and mutations of BRCA genes increases the risk of BC inherent to the genetic cause [140]. This is important data, since obesity or being overweight is one of the most common medical problems in developed countries, and worldwide obesity has more than doubled since 1980, now affecting about 39% of the global population [141]. There are multiple links between obesity and BC. We are going to consider those potentially susceptible to responding to melatonin treatment (Figure 5).

Obviously, the prevention of obesity is the first way to avoid the BC risk related to being overweight. Melatonin should be considered as a tool for obesity management. Various experiments carried out in rats have demonstrated that chronic melatonin consumption prevents the effects of obesogenic diets [142]. Furthermore, in ovariectomized rats, used as a model of menopause, melatonin decreased food intake and partially prevented the increase of BW observed in control animals after suppression of ovarian function [143]. Several clinical trials have confirmed the usefulness of melatonin for obesity treatment. In one of these, a randomized, double-blind, placebo-controlled study in postmenopausal women treated with either melatonin (1–3 mg nightly) or placebo for 1 year, a significant decrease of fat mass was observed, as well as an increase of lean mass in the melatonin group compared to placebo [144]. More recently, in a survey, conducted with thirty obese persons (BMI ≥ 30 kg/m^2^) participants were randomly treated with melatonin (10 mg/day) or placebo for 30 days, combined with caloric restriction. A significant reduction in BW was observed in obese patients treated with melatonin in comparison with those receiving placebo [145]. Interestingly, it has been theorized that the protective effect of exercise by decreasing BW and, consequently reducing the BC risk dependent of this factor, may operate, at least in part, through an increase of melatonin production induced by the exercise [146]. Sleep deprivation has also been considered among the causes of obesity [147,148,149]. The deficiency of melatonin secretion, as a consequence of sleep loss, is among the links between obesity and sleep deprivation [150,151]. There is consistent evidence supporting the beneficial effects of melatonin supplementation for treatment of obesity due to sleep deficit as well as for treating its related complications [151].

In postmenopausal women ovarian synthesis of estrogens is replaced by local synthesis in peripheral tissues including mammary glands. The expression of aromatase, the enzyme involved in the synthesis of estrogens via aromatization of the adrenal androgens, is present in mammary adipose tissue of normal and tumoral glands [152]. One of the most deleterious consequences of obesity in postmenopausal women is the increased aromatase expression, responsible for stimulating the synthesis of estrogens that, in breast tumors, can reach concentrations 10-fold higher than in blood [153]. Additionally, obese postmenopausal women show decreased levels of sex hormone-binding globulin (SHBG) a protein that, in the bloodstream, binds sex steroids and regulates their bioavailability [154,155]. The elevated local concentration of estrogens in mammary tissue of obese women is the cause of tumor initiation and progression [156]. As commented in a previous section of this review, melatonin, due to its SERM and SEEM properties reduces estrogen levels and could be used as a preventive measure for lowering BC risk in obese women [35,36,37,38,39,40,41,42,43,44,45,46,47,48,49,50].

Being overweight or obese is a result of an excessive accumulation of fat in white adipose tissue (WAT) with hyperplasia and hypertrophy of adipocytes, and the development of a state of chronic inflammation of the WAT. In addition to the storing triglycerides, WAT is the source of bioactive molecules globally termed as adipokines, which have been correlated with the development of BC [157,158]. Among the adipokines, decreased levels of adiponectin and increased concentrations of leptin are associated with BC [157,159].

Adiponectin, whose levels are inversely correlated with adiposity, exerts direct growth-inhibitory effects on tumor cells by inhibiting cell proliferation and angiogenesis, and upregulating apoptosis [160]. The levels of this adipokine are lower in BC patients [157,158] and this fact would explain the increased BC risk in postmenopausal obese women [145]. In vitro, adiponectine inhibits proliferation of MCF-7 cells [161]. Several experimental studies in rodents have described a stimulatory effect of melatonin in adiponectin secretion [162,163]. In humans, melatonin (10 mg/day) significantly increased the adiponectin levels, facilitating BW reduction and preventing the oxidative stress in the initial stages of weight loss [145]. These results support the use of melatonin in the treatment of obesity and the reduction of the risk of BC associated with being overweight.

Regarding leptin, its concentration is increased in the obese, reflecting the amount of fat in the body. In mammary glands, leptin is synthesized in preadipocytes, adipocytes and epithelial cells [164]. This hormone, is involved in the normal development of mammary glands and lactation [165], but also stimulates cell proliferation, and invasiveness in breast cancer tissue [166], where its levels are higher than in normal breast tissue [167] The stimulatory effects of leptin on BC growth occurs in different ways including the activation of the ER [158], upregulation of VEGF, an increase in aromatase activity, or increase in telomerase activity [168,169,170,171]. A recent review of the published experimental studies concerning the interrelationship between melatonin and leptin was inconclusive, since both a lack of melatonin effects on leptin secretion as well as melatonin-induced increases or decreases of leptin levels were described. The reviewers attributed these discrepancies to variations in the experimental designs [151]. In postmenopausal women leptin levels were not altered by the daytime administration of melatonin [172]. These facts, contrary to that which happens with adiponectin, do not support any direct effect of melatonin on leptin secretion. However, melatonin’s effects against the mechanisms involved in the leptin-induced carcinogenesis supports its use in reducing the BC risk associated with obesity.

Obese persons become insulin resistant (IR), as a part of the metabolic syndrome (MS). This IR is due to the effects of some of the adipokines such as tumor necrosis alpha, interleukine-6 or resistine [173]. Another hormonal factor that links obesity and breast cancer is the high circulating levels of insulin in obese women, as a consequence of the increased pancreatic synthesis to compensate for their insulin resistance [171]. Insulin increases the proliferation of ER+ MCF-7 cells (but not of ER− breast cancer cells) by increasing the release of growth factors that stimulate mitosis and inhibit apoptosis [171,174]. A meta-analysis of 17 prospective studies carried out in 12 countries concluded that the circulating concentration of IGF-1 is positively associated with the risk of ER+ BC in both pre and postmenopausal women [174]. The usefulness of melatonin for the treatment of MS has been widely demonstrated in animal models [175] as well as in clinical trials showing that administration of melatonin decreases blood glucose levels and decreases IR [144,176,177,178,179].

Recently, it has been shown that the oral antidiabetic drug metformin, used in the treatment of type 2 diabetes, has in vitro antiproliferative effects in different breast cancer cells lines [180], and previously it was observed that patients treated with metformin showed a 31% reduction in the incidence of different cancers including BC [181]. Consequently, metformin is being considered as a drug to aid in BC prevention in obese women with IR [182,183,184]. In this regard, it is remarkable that several experimental studies in animal models of BC have concluded that the association of melatonin and metformin inhibited mammary tumor growth by stimulating apoptosis. These results suggest the possible value of administering melatonin supplements in patients treated with metformin to reduce the incidence of breast cancer [185,186,187].

## 5. Conclusions

In this review we have summarized the evidence in favor of the possible use of melatonin as an adjuvant therapy for individuals with risk factors for BC. One conclusion is that experimental studies provide results which support the value of melatonin as an oncostatic drug for reducing the risk of ER+ breast cancer. The antiestrogenic effects of melatonin (as SEEM and SERM); the additive results of its association with conventional antiestrogenic drugs (tamoxifen, raloxifen, etc), improving their positive effects while mitigating or preventing their unwanted side effects; its antioxidant properties; its effects in obesity and associated metabolic disorders; and its properties as a chronobiotic agent, countering the effects of exposure to LAN, these are all arguments that, together with its lack of toxicity, strongly recommend the realization of clinical trials to evaluate whether this basic knowledge is applicable to programs for reduction of BC risk.
